# Revisiting Traumatic Brain Injury: From Molecular Mechanisms to Therapeutic Interventions

**DOI:** 10.3390/biomedicines8100389

**Published:** 2020-09-29

**Authors:** Abbas Jarrahi, Molly Braun, Meenakshi Ahluwalia, Rohan V. Gupta, Michael Wilson, Stephanie Munie, Pankaj Ahluwalia, John R. Vender, Fernando L. Vale, Krishnan M. Dhandapani, Kumar Vaibhav

**Affiliations:** 1 Department of Neurosurgery, Medical College of Georgia, Augusta University, Augusta, GA 30912, USA; ajarrahi@augusta.edu (A.J.); mobraun@uw.edu (M.B.); Rgupta@augusta.edu (R.V.G.); wilsonma@evms.edu (M.W.); munie@musc.edu (S.M.); jvender@augusta.edu (J.R.V.); fvalediaz@augusta.edu (F.L.V.); kdhandapani@augusta.edu (K.M.D.); 2 Department of Psychiatry and Behavioral Sciences, University of Washington School of Medicine, Seattle, WA 98195, USA; 3 VISN 20 Northwest Mental Illness Research, Education and Clinical Center (NW MIRECC), VA Puget Sound Health Care System, Seattle, WA 98108, USA; 4 Department of Pathology, Medical College of Georgia, Augusta University, Augusta, GA 30912, USA; mahluwalia@augusta.edu (M.A.); pahluwalia@augusta.edu (P.A.); 5 School of Medicine, Eastern Virginia Medical School, Norfolk, VA 23501, USA; 6 College of Medicine, Medical University of South Carolina, Charleston, SC 29425, USA

**Keywords:** neurotrauma, neuroinflammation, excitotoxicity, oxidative stress, apoptosis, edema, brain injury, therapeutic strategies

## Abstract

Studying the complex molecular mechanisms involved in traumatic brain injury (TBI) is crucial for developing new therapies for TBI. Current treatments for TBI are primarily focused on patient stabilization and symptom mitigation. However, the field lacks defined therapies to prevent cell death, oxidative stress, and inflammatory cascades which lead to chronic pathology. Little can be done to treat the mechanical damage that occurs during the primary insult of a TBI; however, secondary injury mechanisms, such as inflammation, blood-brain barrier (BBB) breakdown, edema formation, excitotoxicity, oxidative stress, and cell death, can be targeted by therapeutic interventions. Elucidating the many mechanisms underlying secondary injury and studying targets of neuroprotective therapeutic agents is critical for developing new treatments. Therefore, we present a review on the molecular events following TBI from inflammation to programmed cell death and discuss current research and the latest therapeutic strategies to help understand TBI-mediated secondary injury.

## 1. Introduction

**Figure 1:**
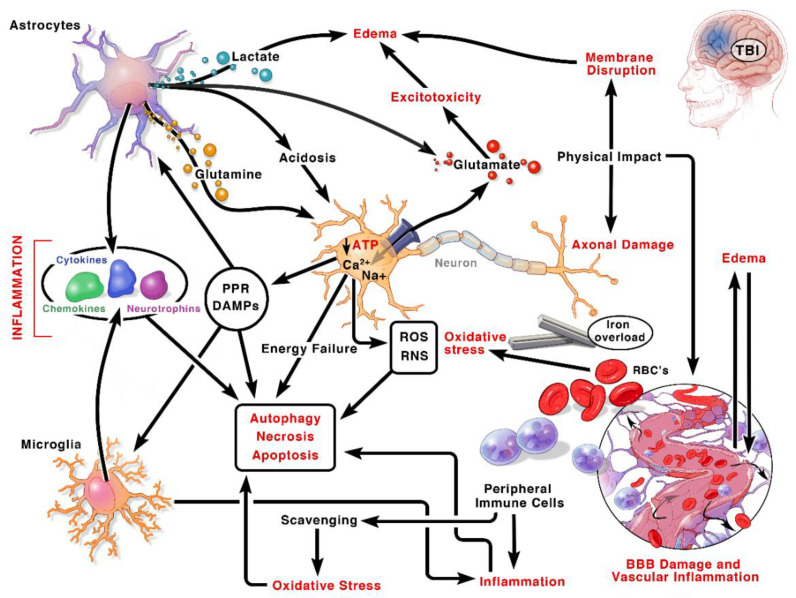
Pathophysiology of TBI. A schematic flow chart of the pathological changes after TBI that lead to acute and chronic neurovascular damage and immune activation. Immediately after the insult neurovascular damage occurs, and large amounts of DAMPs are released causing gliosis and peripheral immune cell infiltration. The initial function of these immune cells is to contain the injury and remove debris and dead cells. However, unregulated immune cells cause enhanced inflammation and injury progression. Furthermore, energy failure, oxidative stress, prolonged inflammation, and excitotoxicity lead to progressive injury with white matter damage and chronic behavioral deficits. Abbreviations: DAMP: Damage associated molecular patterns; PRR: Pattern recognition receptors; ROS: Reactive oxygen species; RNS: Reactive nitrogen species; RBC: Red blood cells; Na^+^: Sodium ion; Ca^2+^: Calcium ion; ATP: Adenosine triphosphate; TBI: Traumatic brain injury.

**Figure 2:**
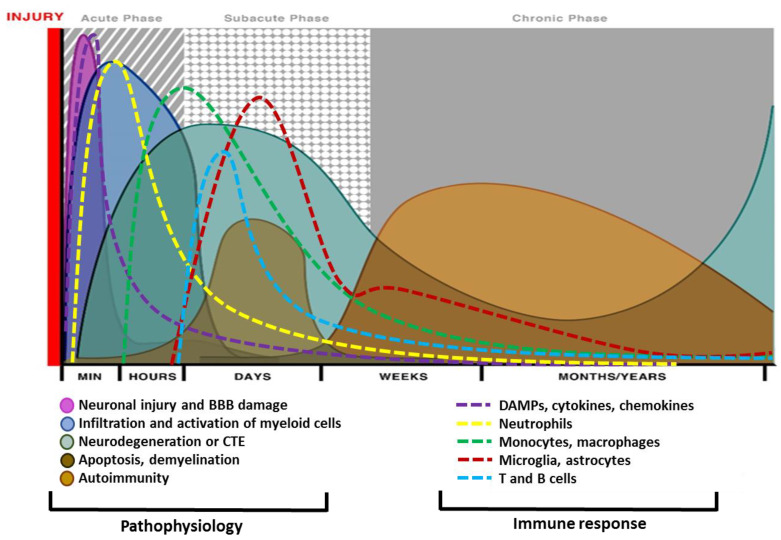
Different phases of traumatic brain injury (TBI) pathophysiology and relative immune response. Mechanical insult leads to acute neuronal injury and blood-brain barrier (BBB) damage, which initiates gliosis and glial injury minutes after TBI and continues for days after injury. Necrotic and apoptotic cell death start immediately after the insult and peak within h to days. Axonal shearing is another event that leads to demyelination and white matter injury. Neurodegeneration, traumatic encephalopathy, and axonal injury may sustain for years after a single TBI. Acute insult and neurovascular damage lead to myeloid accumulation and recruitment of T-cells that last for years and may cause chronic neurodegeneration and neuropathology. Immune cells respond to trauma in a timely manner and a differential pattern of activations has been observed by various studies. An impact to the head leads to cellular damage and results in the rapid release of damage-associated molecular patterns (DAMPs). DAMPs stimulate local cells to release inflammatory mediators such as cytokines and chemokines. These mediators recruit myeloid cells specifically neutrophils as first responders, which phagocytize debris and damaged cells promoting the containment of the injury site. As neutrophil numbers begin to decline, infiltrated monocytes and glia get activated and accumulate around the site of injury to perform further phagocytic or repair functions. Depending on the severity of the brain injury, myeloid cells can recruit T and B cells. T and B cells appear at the sites of brain pathology at later time points in the response (3–7 days post-injury) and may persist for weeks to months. Other abbreviation is as CTE: Chronic traumatic encephalopathy.

Traumatic brain injury (TBI), a leading cause of death and disability, is an international public health concern. An estimated 53–69 million individuals worldwide sustain a TBI annually [1], and up to 2 percent of the population lives with neurological disabilities caused by a TBI [2,3]. TBI occurs when an external mechanical force causes a disruption in normal brain functioning. While commonly discussed as a single clinical entity, TBI embodies a complex and heterogeneous pathology (Figure 1 and Figure 2). As such, comprehensive knowledge of the cellular and molecular events post-TBI remains a long-standing goal of preclinical research, with the hope that this knowledge will spur the expansion of novel therapeutics.

TBI is categorized according to pathophysiology, etiology, and severity, as assessed by neuroimaging and physiological responses. The Glasgow Coma Scale (GCS) is most commonly utilized to define the severity of brain injury in clinical settings, where patients are assessed following initial resuscitation and within 48 h post-injury [4]. A GCS score of 13–15 is classified as mild injury, a score of 9–12 is classified as moderate injury, and a score of <9 is classified as severe injury. Another assessment tool similar to the GCS is the Full Outline of Unresponsiveness (FOUR) score, which can be used in intubated patients and includes an assessment of brainstem function [5].

The pathogenesis of TBI may be divided into two injury-mechanisms: primary and secondary injury. Primary injury entails the direct brain damage that occurs immediately after the impact. The initial injury mechanisms could give rise to extraparenchymal hemorrhages (epidural hematoma, subdural hematoma, subarachnoid hemorrhage, and intraventricular hemorrhage); focal contusions and intraparenchymal hemorrhages; traumatic axonal (focal or diffuse) injury (TAI) due to shearing of WM tracts; and cerebral edema (Figure 1 and Figure 2). Secondary injury mechanisms are also initiated at the moment of the traumatic incident but are believed to continue for many years through a series of cellular, physiological and molecular processes impacting all kinds of cells in the brain. Blood-brain barrier (BBB)-disturbance, excitotoxicity, mitochondrial dysfunction, oxidative stress, inflammation, and cell loss are the principal identified mechanisms orchestrating secondary injury mechanisms [6]. Thus, a pathophysiological and anatomical based classification system for TBI that links the precise pathological mechanisms with the appropriate therapeutic interventions would enhance the translation of therapies from bench to bedside [7]. Therefore, this review provides a synopsis of the main mechanisms of secondary brain damage, along with targeted current and potential neuroprotective therapeutic interventions in preclinical and clinical settings. In the following sections, we will present the historical context for targeting a number of secondary injury pathways. We will discuss the rationale, preclinical evidence, and where appropriate, the translational data in humans. This will provide a segue to why we discuss all these topics, show where we have failed, and perhaps gives a clue why some targets were unsuccessful. This can guide both experimental studies and clinical trial design as we seek efficacy treatments.

## 2. Excitotoxicity

Excitotoxicity is a pathological process where accumulation of excitatory neurotransmitters, usually glutamate and over-activation of their receptors (NMDAR), causes BBB damage, loss of neuronal membrane integrity, edema, and cell loss after TBI [8]. Studies involving the administration of membrane-resealing polymers following controlled cortical impact (CCI) reported reduced BBB permeability and cerebral damage, and improved functional recovery [9,10] but failed to rescue degenerating cells [11]. This may suggest that resealing of these membranes prevents further alterations of the membrane but did not rescue degenerating cells that were already damaged by the initial episode of TBI-induced excitotoxicity. Similarly, persistent elevated glutamate in cerebral tissue and CSF link with TBI severity in patients [12,13]. Although NMDAR antagonism mitigated TBI-induced neurological damage in rodents [14], global NMDAR antagonists showed side effects and were associated with poor therapeutic windows [15]. Thus, revelation of the mechanisms linking glutamate excitotoxicity, NMDAR activation, and consequent neurological damage, may offer a roadmap to improve neurological outcomes without any adverse effect.

### 2.1. Glutamate

Glutamate, the principal excitatory neurotransmitter, is essential for normal brain function; however, metabolic perturbations occurring immediately after neurotrauma result in loss of ATP production and subsequent failure of neuronal Na^+^-K^+^ ATPases. These changes disrupt the homeostatic balance of the electrochemical gradient, causing intracellular sodium accumulation and neuronal depolarization to exacerbate release of synaptic glutamate. In addition, mechanical stretching of neuronal membranes induces micropore formation to aggravate intracellular sodium influx. This ionic shift exacerbates the opening of voltage-gated calcium channels and neuronal depolarization to further the excessive synaptic release of glutamate. Microdialysis studies have shown that increased levels of extracellular glutamate post-TBI correlated with the severity of injury, while elevated glutamate levels in CSF and brain tissues correlated with worse outcomes after clinical TBI [12,16,17,18,19].

### 2.2. Glutamate Receptors

Glutamate receptors are present on membranes of neurons and glial cells both at synaptic and extra-synaptic regions. There are two types of glutamate receptors: (GluR)-ionotropic (iGluR) and metabotropic (mGluR). When glutamate binds to iGluRs (ligand-gated nonselective cation channels), it activates the ion channels, and when it binds to mGluRs (G protein-coupled receptors), it either upregulates or downregulates signal transduction pathways. iGluRs are in turn categorized into four subtypes, including N-methyl-D-aspartate receptors (NMDAR), kainate receptors (KAR), α-amino-3-hydroxy-5-methyl-4-isoxazolepropionic acid receptors (AMPAR), and delta receptors. Glutamate increases intracellular calcium primarily through activation of postsynaptic ionotropic receptors, such as NMDARs [20,21]; however, KARs and calcium-permeable AMPARs also may add to elevated intracellular calcium. Excessive inflow of calcium activates phospholipases, endonucleases, and proteases (calpains), which then precipitate neuronal cell loss via a process deemed excitotoxicity. In addition, GluRs, which are particularly sensitive to mechanical injury, may also contribute to delayed depolarization and injury [22].

#### 2.2.1. Synaptic and Extrasynaptic NMDARs

Based on their location, NMDARs can exert opposing effects. Although stimulation of synaptic NMDAR upregulates brain-derived neurotrophic factor (BDNF) and cAMP response element-binding protein (CREB) activity to promote neuronal survival, extrasynaptic NMDAR activation leads to excitotoxicity and neuronal cell death. This occurs via promotion of a CREB shut-off pathway and inhibition of BDNF gene expression. Specific targeting of NMDARs based on their location could pave the way towards more effective neuroprotective therapies [23,24,25].

#### 2.2.2. NMDAR Subunits

A more comprehensive analysis of NMDARs shows that they can be made up of seven variable subunits, including a GluNR1 subunit, four GluNR2 subunits (GluN2A, GluN2B, GluN2C, or GluN2D), and two GluNR3 subunits (GluNR3A and GluNR3B). NMDARs are heterotetramers with a strictly regulated subunit composition [26]. Further, extra-synaptic and synaptic NMDARs have different subunit compositions which could be a reason for their opposing cellular effects. The majority of extrasynaptic NMDARs are composed of NR1/NR2B subunits, while synaptic receptors also contain NR2A subunits. Moreover, NMDARs at immature sites markedly contain NR1/NR2B subunits, while NMDAR composition is more diverse at mature sites and switches from NR2B to NR2A on synaptic maturation, termed as NR2B to NR2A developmental switch [27]. Recently, it has been reported that higher expression of the NMDAR subunit NR2A protected neuronal connectivity in the injured brain, while activation of NR2B-containing NMDARs contributed towards loss of connectivity, suggesting a potential role for NMDARs in the restructuring of the neuronal network post-TBI [28].

#### 2.2.3. Therapeutic Strategies Targeting NMDARs

Many therapeutic interventions have been designed based on targeting NMDARs, including NMDAR antagonists, NMDAR subunit inhibitors, and partial agonists of glycine/NMDAR.

### NMDAR Antagonists

Administration of NMDAR antagonists (e.g., MK-801) improved outcomes after experimental TBI [29,30,31,32]; yet clinical trials exploring the efficacy of NMDAR antagonists were halted due to poor efficacy, major side effects, poor drug efficacy, restricted therapeutic windows, and interference with normal synaptic transmission. These disappointing results, which suggest limited utility of broad-spectrum NMDAR antagonists after acute brain injury, illustrate the translational challenges involved in limiting the detrimental effects of glutamate and suggest the need for alternative approaches [15,33,34,35].

MK-801: Rats treated with MK-801 prior to injury demonstrated enhanced cognition and axo-dendritic integrity [30], and co-application of MK-801 with other NMDAR antagonists had additive neuroprotective effects [31]. Treatment with MK-801 prior to injury, significantly attenuated neurological deficits; however, MK-801 had minimal influence on neurologic scores when was administered post-injury [32].

Memantine: Intraperitoneal treatment of memantine (10 and 20 mg/kg), a non-competitive NMDAR antagonist, immediately after TBI inhibits neuronal death in rats [36]. Memantine (1–10 μM), when applied to rat hippocampal neurons in vitro, inhibited extrasynaptic NMDAR-induced currents while largely sparing synaptic activity [37]. In a randomized controlled trial of moderate TBI patients, enteral administration of memantine (30 mg twice daily for 7 days) post-injury resulted in substantial improvement in GCS scores at 3 days and significant reduction in neuronal damage at 7 days, as evident from reduced serum levels of neuron-specific enolase (NSE) [38].

Ketamine: In a moderate TBI rat model, administration of ketamine, a non-competitive NMDAR antagonist, at a sub-anesthetic dose (10 mg/kg daily) for 7 days resulted in protection of neuronal dendrites and spines, attenuation of post-traumatic neuroinflammation, and thus, improved neurobehavioral outcomes [39]. Similarly, a significant association between ketamine treatment and reduced spreading depolarization events have been reported in TBI, SAH, and hemispheric stroke patients. Spreading depolarizations are linked with neuronal damage and poor outcome [40].

Magnesium: Magnesium can bind to NMDARs, blocking the passage of ions through the channel, and can therefore be utilized to augment neuroprotection. A study in rats reported that magnesium deficiency worsened TBI outcomes while magnesium administration improved neurological outcomes and mortality after TBI [41]. A Cochrane systematic review including three randomized controlled trials (RCTs) published in 2008, found no beneficial role for magnesium treatment in acute TBI patients in terms of improving neurological outcomes or mortality and therefore did not support its use [42]. Another methodical review of the use of magnesium sulfate in acute TBI management, including eight RCTs published in 2015, also found no significant improvement in mortality but did report a nonsignificant improvement in GOS and GCS scores with magnesium therapy [43].

### NMDAR Subunit Inhibitors

Ifenprodil: Ifenprodil, a selective inhibitor of NR2B subunit, can differentially suppress extrasynaptic NMDARs, and therefore could attenuate cell death cascades [44]. Similarly, Ifenprodil treatment mitigated brain edema and reduced injury volume in a CCI model of rats [45]. In an in vitro model of TBI, Ifenprodil reduced NMDA-activated currents but failed to limit fluid shear stress-induced Ca^2+^ influx in primary rat astrocytes [46].

Ro25-6981: TBI modifies NMDAR expression and functioning. For example, moderate TBI in rats caused rapid recruitment of NR2B to membrane rafts successively inducing autophagy. Ro25-6981, a selective inhibitor of NR2B, markedly mitigated autophagy [47]. Activation of NR2B-containing NMDARs may be further involved in the insertion of calcium-permeable AMPARs (CP-AMPARs) in an in vitro model of TBI, further worsening the intracellular calcium overload [48]. Stretch injury of cultured cortical neurons resulted in enhanced extrasynaptic current transmission facilitated by NR2B-units of NMDARs, with no marked variations in synaptic transmission through NMDAR [49]. In addition, either Ro25-6981 or memantine treatment barred this injury-induced increase in CP-AMPAR activity [49]. Further, administration of Ro25-6981 (6 mg/kg, i.p.), attenuated edema post-TBI in mice and limited the NMDA-induced excitotoxicity and release of HMGB1 from injured cortical neurons in vitro [14].

Traxoprodil (CP-101,606): Intravenous (IV) infusion of traxoprodil in patients, another NR2B antagonist, for up to 72 h post-injury was reported to be safe and well-tolerated in all cases of TBI [50,51]. Although in an RCT where severe TBI patients were given a 72-h infusion of traxoprodil within 8 h post-injury, improvements in the dichotomized Glasgow Outcome Scale (dGOS) at 6 months and mortality rate were noticed; however, this improvement was not significant [52].

Temporal alteration of NMDARs and the partial agonist D-cycloserine (DCS): TBI in rodent models, leads to dynamic changes in NMDARs with early hyperactivation followed by weeks of functional loss. Subacute administration (24 to 72 h post-TBI) of DCS, a partial agonist of glycine/NMDARs, upregulated BDNF expression, restored impaired hippocampal long-term potentiation, and enhanced recovery of neurobehavioral and cognitive functions in mice [53]. The complex role of NMDARs in TBI pathophysiology adds to the translational challenges faced by therapeutic interventions targeting this mechanism.

### 2.3. Postsynaptic Density Protein 95 (PSD-95) and PSD-95 Inhibitors

PSD-95 is a membrane-associated guanylate kinase (MAGUK) that interacts with NMDARs, AMPARs, and potassium channels and plays a role in synaptic plasticity [54]. PSD-95 combines the NR2B subunit of NMDARs with neuronal nitric oxide synthase (nNOS), to form a complex NMDAR/PSD-95/nNOS, and adds to neurotoxicity [55]. Interestingly, inhibition of nNOS by disturbing NMDAR-PSD-95 interactions prevented nitration of protein and cell death in vitro [56]. ZL006, an inhibitor of nNOS-PSD95 interaction, prevented neuronal apoptosis and improved sensorimotor and cognitive outcomes in rodents [57]. Similarly, disruption of NMDAR-PSD-95 interaction by a synthetic peptide (now known as NA-1) blocked excitotoxicity in cultured neurons, limited ischemic cerebral damage, and improved neurological function in rats without altering calcium influx or synaptic activity [58]. The strategy appears to be very promising as inhibition of this interaction between PSD-95 and NMDAR-mediated neurotoxic signaling pathways has demonstrated reduced infarct size and improved outcomes after stroke in macaques [59]. Further, NMDAR-PSD-95-nNOS complex inhibitor, known as NA-1, has been shown to treat ruptured cerebral aneurism, reduce ischemic brain damage, and improve neurological scores, and has become the first stroke therapy to demonstrate efficacy in humans (NCT00728182) after initial results in primates [60,61,62]. In addition, two phase III clinical trials (NCT02315443; NCT02930018) have been completed in acute stroke and awaiting publication. Interestingly, TBI activated endoplasmic reticulum-associated PKR-like ER kinase (PERK) which phosphorylates CAMP response element-binding protein (CREB) and PSD-95, resulting in reduced brain-derived neurotropic factor (BDNF) and PSD-95 in the injured cortex. However, either PERK inhibitor GSK2656157 or overexpression of kinase-dead mutant of PERK (PERK-K618A) in primary neurons rescued loss of dendrites and memory in mice [63]. Further, a rodent model of CCI showed loss of PSD-95 with loss of neuronal NeuN in contused cortex and directly correlated with behavioral abnormalities [64]. UCCB01-147 [also known as Tat-NPEG4(IETDV)(2), (Tat-N-dimer)], a dimeric PSD-95 inhibitor, was observed to be neuroprotective in an experimental stroke model [65] but failed to demonstrate those beneficial effects in experimental TBI [66]. Therefore, it can be argued that PSD-95 alone or with other effector proteins may provide a potential clinical therapeutic target to improve memory and learning deficits but must be translated carefully for better results post-TBI.

### 2.4. mGluR2 Receptors and Gap Junctions

Recently, it was reported that mGluR2 receptors in a fluid percussion TBI model once activated, upregulated gap junction protein expression, suggesting a possible role for mGluR2 and gap junctions in secondary brain injury [67]. Gap junctions play critical roles in neuronal differentiation and circuit formation in the developing CNS and allow for the passage of ions, small molecules, and secondary messengers (Ca, IP3, cAMP, etc.) in the adult brain [68,69]. However, upregulated gap junctions in insulted brain may enhance the secretion of pro-apoptotic factors and secondary messengers such as Ca^2+^ to add to cell death. Therefore, mefloquine, a gap-junction blocker may be a valuable therapeutic tool in mitigating TBI-induced excitotoxicity.

### 2.5. Glutamate Transporters

The solute carrier 1 (SLC1) family of neurotransmitter transporters includes a five-member family of excitatory amino acid transporters (EAAT) that mediate the rapid uptake of synaptic glutamate via a process coupled to ion gradients [70]. Amongst the EAAT, EAAT1 [Glutamate Aspartate Transporter (GLAST)] (human/rodent homolog) and EAAT2 [Glutamate Transporter 1 (GLT-1)] are essential for glutamate clearance related to neurotransmission, whereas EAAT3, EAAT4, and EAAT5 exhibit less prominent parts in regulating neuronal excitability [70]. In particular, EAAT2 is expressed in glia and mediates 95 percent of glutamate uptake in CNS [71]. Notably, when ionic gradients are lost, sodium-glutamate transporters can reverse the transport direction to secrete a high amount of glutamate [72]. Given that excessive glutamate is associated with excitotoxicity, targeted enhancement of EAAT2 may circumvent the issues associated with administration of NMDAR antagonists. Postmortem analysis of human brains obtained after TBI showed lower expression of the glial glutamate transporter EAAT2, which might have impaired reuptake of extracellular glutamate and thus, have led to excitotoxicity [73,74]. Similarly, lowered EAAT2 expression inversely correlated with CSF glutamate levels up to 7 days post-injury in CCI model of rodents [75,76].

MS-153 (GLT-1 activator): Functionally, administration of GLT-1 antisense oligodeoxynucleotides exacerbated hippocampal injury and increased mortality after TBI [76], whereas acute administration of (R)-(-)-5-methyl-1-nicotinoyl-2-pyrazoline (MS-153), a GLT-1 activator, decreased neurodegeneration and attenuated calpain activation in cortical and hippocampal tissue for up to two weeks after fluid percussion injury. While these latter findings warrant further exploration of GLT-1 activators, MS-153 upregulated GLT-1 in the naïve brain but not after brain injury in rats, suggesting that mechanisms independent from GLT-1 may mediate the observed beneficial effects [77]. Future studies incorporating more selective pharmacological activators and genetic overexpression approaches will provide clarity regarding the translational potential of targeting GLT-1 after TBI.

### 2.6. Blood Glutamate Scavengers

Glutamate transporters located on brain capillary endothelial cells facilitate brain-to-blood efflux of glutamate and play a role in glutamate homeostasis. When glutamate concentrations are kept low in blood, this results in a larger concentration gradient of glutamate and enhances its brain-to-blood efflux [78,79]. Glutamate levels in the blood might be reduced via two enzymes: glutamate-oxaloacetate transaminase (GOT or AST) (l-glutamate + oxaloacetate ⇌ α-ketoglutarate + l-aspartate) and glutamate-pyruvate transaminase (GPT or ALT) (l-glutamate + pyruvate ⇌ α-ketoglutarate + l-alanine). The two serum enzymes (SGOT and SGPT) and co-substrates (oxaloacetate and pyruvate) may potentially act as glutamate scavengers. In agreement, treatment with co-substrate, oxaloacetate, and pyruvate resulted in a reduction of glutamate in blood and enhanced neuronal survival and neurological outcomes in experimental TBI studies [80,81]. In addition, recombinant GOT1 has also shown promising results in TBI [82], and ischemic stroke [83,84]. Of note, riboflavin (Vitamin B2) was found to be a potent scavenger of blood glutamate, resulting in reduced infarct size after ischemia [85]. Similarly, Hoane and colleagues found that riboflavin significantly reduced sensorimotor and cognitive impairment, reduced edema, and astrogliosis after TBI [86] Furthermore, a double-blind, randomized phase IIb clinical trial in stroke patients also demonstrated riboflavin significantly scavenged glutamate in patient blood [85]. Taken together, these studies suggest that therapies utilizing blood glutamate scavenging may be promising therapeutic avenues for reducing glutamate-induced excitotoxicity.

### 2.7. GABAergic Excitotoxicity

Although glutamate is a major player in excitotoxicity, elevated concentrations of other neurotransmitters have also been detected in the extracellular space of injured brains. These other neurotransmitters, such as GABA, may also aid in excitotoxicity in both specific and distant cell populations. A microdialysis study in experimental open-skull weight drop TBI, showed elevated GABA in the cortical extracellular space [87]. Similarly, hippocampal cell loss has been reported in multiple experimental models of TBI and may correlate with neurocognitive deficits that occur in TBI patients [88,89]. Immature neurons in the adult hippocampal sub-granular zone express voltage-gated channels similar to their embryonic equivalents and may be depolarized by GABAergic activity via chloride gradient reversal [90,91]. Since selective necrotic cell loss among immature adult-born neurons has been reported after CCI injury; therefore, the properties of these immature neurons may be relevant to pathophysiology in TBI [92,93,94]. However, the mechanism behind this particular cellular selectivity for necrosis among immature cells is still known. GABAergic excitotoxicity also contributes to neuronal loss as exposure of isoflurane to the immature pyramidal cells in culture increased intracellular calcium and led to cellular death [95], suggesting that intracellular calcium overload may add to immature neuronal death. These studies, coupled with the increased extracellular GABA concentrations as observed following TBI [87], may implicate an unexplored role for GABAergic excitotoxicity post-TBI. Understanding the mechanisms of GABAergic excitotoxicity in the post-traumatic brain may be valuable therapeutically, as several GABA antagonists, approved by the U.S. Food and Drug Administration (FDA), are available. In terms of treating more canonical glutamatergic excitotoxicity, it may be argued that antagonists to GluR or inhibitors that block the release of glutamate inhibitors (e.g., lamotrigine) exert beneficial effects not only through inhibition of either GluR or glutamate, but also by minimizing the neuronal metabolism. However, given the contradictory findings of clinical trials utilizing magnesium sulfate as an NMDAR antagonist in acute stroke patients [96,97], a more comprehensive knowledge of excitotoxicity following TBI, including non-canonical mechanisms (such as GABAergic excitotoxicity), is critical for developing effective therapeutic strategies. Further, excitotoxicity and as a result, influx of excessive calcium into cells lead to oxidative stress, mitochondrial dysfunction, activation of Nox family member, and oxidation of cellular biomolecules such as lipids, proteins, and DNA. Furthermore, excessive amount of intracellular calcium activates several proteases and phospholipases, and thus, mediates degradation of cellular proteins and lipids, and enhances ROS production, and contributes significantly to secondary injury post-TBI [98,99].

## 3. Oxidative Stress

TBI results in cerebral circulation dysfunction, microvascular impairment, and moderate hypoxia [100,101]. Although primary traumatic injury occurs as a result of the physical impact, tissue injury is amplified by secondary injury mechanisms. Oxidative damage is unambiguously one of the most confirmed “secondary injury” pathways observed in TBI. The brain is very sensitive to free radical-mediated damage because of the presence of abundant polyunsaturated lipids and a high rate of endogenous oxidative metabolism. Therefore, a balance between oxidant production and antioxidant machinery is essential for normal functioning of the brain.

### 3.1. Oxidant-Antioxidant Balance

The reperfusion of blood circulation after trauma ensures the survival of neurons but also elevates the generation of free radicals and reactive oxygen species (ROS) [102,103,104]. The generated free radicals, such as hydrogen peroxide, superoxides, nitric oxide (NO), etc., also cause excitotoxicity and impair the metabolic activity of cells. Further, superoxide radicals generated due to catalytic activity after TBI react with NO to form another potent oxidant peroxynitrite, which impairs cerebrovascular function [105,106]. The ROS possesses an unpaired electron and thus, readily binds to different macromolecules such as protein, nucleic acid, or lipid to cause damage. The endogenous antioxidant system comprises of glutathione (GSH), and various enzymes (i.e., glutathione reductase (GR), glutathione-S-transferase (GST), glutathione peroxidase (GPx), catalase (CAT), superoxide dismutase (SOD), and uric acid) neutralizes these ROS, preventing the oxidation of macromolecules. In a normal brain, oxidants and antioxidants exist in equilibrium; however, the unwarranted production of ROS following brain injury overburdens the efficiency of the endogenous antioxidants and shifts the equilibrium towards oxidants by depleting endogenous antioxidants. This disrupted balance increases membranous lipid peroxidation, oxidation of proteins, DNA break, and inhibition of the mitochondrial respiration, which ultimately throws cells into apoptosis or necrosis [107]. Overall, increased oxidants and reduced activity of antioxidant defense systems may contribute toward the pathogenesis post-TBI.

### 3.2. Superoxide Radicals and Superoxide Scavengers

Kontos and colleagues demonstrated an acute increase in brain microvascular superoxide radical (O_2_•^−^) contents as a result of compromised autoregulatory function after fluid percussion injury [108,109]. Within an injured brain, several possible sources of O_2_•^−^ may be operating from the very first minute of impact to a few h post-injury, including the arachidonic acid-prostaglandin cascade, oxidation of leaked hemoglobin and biogenic amines (e.g., norepinephrine, dopamine, 5-hydroxytryptamine), xanthine oxidase activity, and mitochondrial leakage. At later time points, activated microglia and infiltrating neutrophils and macrophages also provide additional sources of O_2_•^−^ Superoxide O_2_•^−^ is less reactive to biological substrates than hydrogen peroxide (H_2_O_2_). Once formed, O_2_•^−^ undergoes dismutation to form H_2_O_2_ in a reaction that is catalyzed by SOD: O_2_•^−^ + O_2_^−^ + 2H^+^ → H_2_O_2_ + O_2_ [110]. The H_2_O_2_ formed is altered to water by peroxidases, such as Gpx and peroxiredoxin, or is dismuted to water and oxygen by CAT. Both CAT and GPx enzymes are abundant in the brain, though the latter has a sevenfold greater activity [111]. In the absence of the antioxidant system, as in TBI, O_2_•^−^ actually exists in equilibrium with hydroperoxyl radicals (HO_2_•): O_2_•^−^ + H^+^ → HO_2_•, which is a considerably more powerful oxidizing or reducing agent [112]. O_2_•^−^/HO_2_• cause the pH to fall into acidic ranges (i.e., tissue acidosis), fueling an equilibrium shift in favor of HO_2_•, which is much more reactive than O_2_•^−^, particularly toward lipids.

SOD and polyethylene glycol (PEG)-conjugated SOD (PEG-SOD): In humans, the three forms of SOD are SOD1 (Cu/Zn-SOD), SOD2 (Mn-SOD), and SOD3 (Cu/Zn-SOD), which are respectively sited in the cytoplasm, mitochondria, and outside the cell. In cats, the administration of SOD reverses the microvascular dysfunction post-TBI [113]. Transgenic mice overexpressing human Cu/Zn SOD activity have reduced acute injuries, prevented brain edema, and inhibited BBB permeability following TBI [114,115]. Studies using both Cu/Zn-SOD and Mn-SOD transgenic and knockout mice have further solidified the protective role of these enzymes against head trauma [115,116,117,118,119]. In a randomized controlled phase-II trial with more metabolically stable PEG-SOD (2000–10,000 U/kg intravenously administered 4 h post-TBI), the higher doses (5000 and 10,000 U/kg) decreased the duration when ICP > 20 mm of Hg. There was a statistically significant improvement in patient outcomes measured using the Glasgow Outcome Scale (GOS) at 3 and 6 months post-injury between patients who received placebo and those who received 10,000 U/kg PEG-SOD [120]. However, a subsequent larger phase-III randomized trial with higher doses of PEG-SOD (10,000 or 20,000 U/kg within 8 h post-TBI failed to show significant improvement in neurological outcomes or patient survival [121]. Nevertheless, it is imperative to shed light on a 4-h difference in the time of administration of PEG-SOD post-injury between these two trials.

OPC-14117: The superoxide radical scavenger “OPC-14117” reduced cortical damage and improved neuronal survival and cognitive functions following CCI in rats [122]. A controlled randomized trial assessing the safety of OPC-14117 (240 mg per day) in HIV-associated cognitive impairment found it to be tolerable and resulted in improvement of clinical global impression scale scores and nonsignificant enhancement of cognitive test scores [123]. In light of the neuroprotective effects of OPC-14117 in this preclinical TBI study and the safety of use in humans, the potential benefits of OPC-14117 treatment after TBI are worth investigating.

### 3.3. Iron, Hydroxyl Radicals, and Iron Chelators

The abundance of iron in the CNS makes it vulnerable to oxidative insult [124]. Under normal circumstances, iron is maintained in a non-catalytic state by plasma transferrin and intracellular ferritin [110]. However, in the event of tissue acidosis, when pH falls below 6, both proteins readily release their iron into the traumatized brain parenchyma. Further, hemorrhage occurs as a result of mechanical impact provides an obvious pool of iron released from hemoglobin via interaction with H_2_O_2_ or lipid hydroperoxides (LOOH) at acidic pH [125,126]. Once released into the brain parenchyma, iron actively catalyzes Fenton’s reaction to generate ROS [110]. Free iron or iron compounds participate in production of ROS in two ways: First, autoxidation of Fe^2+^ produces O_2_•^−^ [110]: Fe^2+^ + O_2_ **→** Fe^3+^ + O_2_•^−^ and/or secondly, oxidation of Fe^2+^ by H_2_O_2_ gives hydroxyl radicals (•OH) via Fenton’s reaction: Fe^2+^ + H_2_O_2_ **→** Fe^3+^ + •OH + OH^−^.

Deferoxamine and dextran-conjugated deferoxamine: Experimental TBI studies of deferoxamine (iron chelator) treatment in rodents have shown neuroprotective effects [127,128]. However, IV infusion of deferoxamine may cause profound hypotension, but binding of deferoxamine to dextran may alleviate this effect as low dose dextran-conjugated deferoxamine following TBI improved neurological outcomes as compared to the deferoxamine group alone [129].

N,N’-Di(2-hydroxybenzyl)ethylenediamine-N,N’-diacetic acid monohydrochloride (HBED): HBED is an iron chelator, can cross the BBB, and has a relatively longer half-life compared to that of deferoxamine [130]. HBED treatment resulted in reduced cortical damage and restored neurological functions in mice post-TBI [131].

### 3.4. Nitric oxide Synthase (NOS) and NOS Inhibitors

NOS catalyzes l-arginine to give NO and citrulline at the expense of NADPH. The three NOS isoforms [neuronal (nNOS or NOS1), inducible (iNOS or NOS2), and endothelial (eNOS or NOS3)] are acutely upregulated in rats following TBI, with levels peaking at 6 to 12 h post-injury and then declining. eNOS is expressed solely in endothelial cells, nNOS predominantly in neurons but also in polymorphonuclear cells, and iNOS in immune cells such as myeloid cells [132]. Subsequent to injury, an upsurge of NO occurs, possibly because of the hyperactivity of iNOS, and the inhibition of iNOS could be neuroprotective [133]. Clinically, NO levels can be assessed indirectly through CSF measurement of the end products of NO metabolism, and a peak concentration is found at 1–3 days following TBI [134,135,136].

nNOS, NG-nitro l-arginine methyl ester (l-NAME), and 7-nitro indazole (7-NI): Both l-NAME (nonselective NOS inhibitor) and 7-NI (nNOS inhibitor) were reported to exert neuroprotection only when administered within an hour of injury in mice, indicating a narrow therapeutic window post-TBI. L-arginine, the physiological precursor of NO, when co-administered with NOS inhibitors reverses their protective effects [137]. Pretreatment with l-NAME or 7-NI reduced NOS activity after FPI in rat, while 7-NI also improved neurobehavioral outcomes post-TBI [138]. However, another FPI model of TBI study in rats found no beneficial role of l-NAME administration post-TBI with regards to mortality, and pretreatment leads to prolonged hypertensive episodes and increased mortality [139].

eNOS and l-arginine: TBI-induced upregulation of eNOS plays a beneficial role by maintaining cerebral blood flow (CBF) post-head injury. Administration of l-arginine post-TBI in rats activated eNOS, improved CBF, and attenuated neurological deficits without altering cerebral perfusion pressure (CPP) [140]. Similarly, eNOS-deficient mice subjected to CCI have shown lower CBF at the cortical injury site compared to wild-type mice, and l-arginine did not improve the CBF nor the contusion volume in eNOS-deficient mice [141]. In humans, microdialysates from severe TBI patients showed elevated levels of NO metabolites in the first 24 h post-injury followed by a gradual decline over 5 days. There was also a significant direct relationship between the concentration of NO metabolites and regional CBF [142]. In severe TBI patients, l-arginine administration at 48 h post-injury had a better response in improving internal carotid artery flow volume than at 12 h following brain injury [143]. The above findings further emphasize the importance of determining the effective therapeutic window for drug administration following brain injury. In humans, the NOS3 (eNOS) gene, located at 7q35–36, has several allelic variations and patients having the −786C allele showed lower CBF values than other severe TBI patients. Thus, genetic makeup may be a potential contributing factor to the variability in TBI patient outcomes [144].

**Statins:** Statins are HMG-CoA reductase inhibitors with proven neuroprotective activity in experimental TBI studies through targeting of multiple secondary injuries [103]. More precisely, statins upregulate eNOS, have a palliative effect on cerebral autoregulation (CA), and improve stroke outcomes [145].

iNOS and iNOS inhibitors (Aminoguanidine (AG), L-NIL and 1400W): iNOS is an inducible type of NOS which is stimulated by injury-induced stimuli, such as inflammatory modulators, ROS, etc. [146,147,148,149]. In rats exposed to FPI, intraperitoneal injection of 100 mg/kg aminoguanidine (AG) twice daily for 3 days reduced the total cortical necrotic neuron counts but not the contusion volume [150]. Blast induced-TBI in rats showed that those receiving AG either prophylactically or after the injury performed better on neurobehavioral tests and had reduced cortical neuron degeneration compared with those receiving saline injection [151]. In an FPI model of brain trauma in rats at 6 h after injury, the following 3 iNOS inhibitors were given at 6 h post-injury: aminoguanidine (AG; 100 mg/kg intraperitoneally), l-N-iminoethyl-lysine (l-NIL; 20 mg/kg intraperitoneally), or N-[3-(aminomethyl)benzyl]acetamide (1400W; 20 mg/kg subcutaneously). All three improved neurofunctional outcomes, but AG also reduced brain edema [152].

Ronopterin (also termed 4-amino-tetrahydrobiopterin or VAS203): In a phase IIa RCT with moderate to severe TBI subjects (NO Synthase inhibition in traumatic brain injury, NOSTRA), patients were given various IV infusion doses (15–30 mg/kg) of ronopterin, a NOS inhibitor. Ronopterin treatment showed no marked alteration in ICP, CPP, or brain metabolism. Other than a transitory acute kidney injury in half of the patients receiving the highest dose, no other toxic side effects were reported. Additionally, ronopterin had a neuroprotective role shown by significant improvement of extended Glasgow Outcome Scores (eGOS) at 6 months [153]. These positive results lead NOSTRA trial into phase III which is current and whose study protocol has been published [154].

### 3.5. Peroxynitrite and Peroxynitrite Scavengers

Peroxynitrite is formed by the reaction of superoxide and NO radicals, which contributes to impaired cerebral vascular reactivity after TBI [105,106]. Peroxynitrite interacts with DNA, proteins, and lipids via oxidizing or radical-mediated mechanisms. In addition, it reacts with tyrosine residues of proteins to yield nitrotyrosine and impairs activity [155].

Penicillamine and penicillamine methyl ester: The thiol-containing compound penicillamine and the more brain-permeable penicillamine methyl ester are sulfhydryl-based scavengers of peroxynitrite. Both of these compounds have improved early neurological recovery in TBI mice. Although penicillamine remains mainly within the cerebral microvasculature, it showed greater neurological recovery than highly penetrable penicillamine methyl ester, highlighting the significance of early scavenging of intravascular peroxynitrite [156].

Tempol and α-phenyl-N-tert-butyl-nitrone (PBN): The peroxynitrite radical scavengers tempol [157,158,159] and PBN [160] have demonstrated neuroprotective activity in experimental TBI and could be good candidates to minimize nitrosative stress.

### 3.6. Lipid Peroxidation (LP) and LP Inhibitors

Free radical-mediated LP is an extensively studied mechanism of oxidative injury in TBI [161]. The brain cell membrane is abundant in polyunsaturated fatty acids e.g., arachidonic acid (AA), which is extremely susceptible to •OH-induced peroxidation. LP starts when a radical species, such as •OH, extracts hydrogen from an allyl group (AA + R• **→** AA• + RH), converting this allylic carbon into an “alkyl” radical (AA•). During the propagation stage, the resulting alkyl radical binds with a molecule of oxygen to form a lipid peroxyl radical (AA-OO•; AA• + O2 → AA-OO•). The peroxyl radical then reacts with a neighboring AA within the membrane and gains its electron to generate a lipid hydroperoxide (AA-OOH) and a resultant alkyl radical (AA•; AA-OO• + AA → AA-OOH + AA•). This cycle of generation of alkyl radicals is continuous and compromises cellular and sub-cellular membranous integrity. Finally, in the termination step of the LP, lipid radials react with another radical, giving rise to highly reactive, potentially neurotoxic aldehydes known as carbonyls. The neurotoxic aldehydes, 4-hydroxynonenal (4-HNE), and 2-propenal (acrolein) bind to basic amino acids (arginine, histidine, and lysine) and sulfhydryl-containing cysteine residues in cellular proteins, and alter their conformation and function.

Tirilazad (lazaroid, 21-aminosteroid, a LP inhibitor): Previously, tirilazad has been shown to enhance neurological recovery and survival in experimental TBI [162,163,164]. In a multicenter phase III study, moderate-severely injured patients, treated with tirilazard mesylate (10 mg/kg intravenous), starting within 4 h post-injury and repeated for every 6 h up to 5 days, did not show significant GOS on recovery/survival at 6 months. However, a post hoc analysis of data discovered that tirilazad lowered mortality rates in male TBI patients with accompanying traumatic subarachnoid hemorrhage (tSAH) [165].

U83836E: U83836E is a very effective second-generation lazaroid with a unique structure giving it the ability to scavenge lipid peroxyl and to inhibit LP. Further, U83836E also showed the ability to preserve mitochondrial respiratory function in rodents post-TBI [159].

LP carbonyl (4-HNE or acrolein) scavengers

Hydralazine: Despite being shown to have a good ability to scavenge LP carbonyls, hydralazine is a powerful vasodilator exacerbating hypotension in TBI and therefore, is not recommended post-TBI [166,167].

Phenelzine: Phenelzine, a monoamine oxidase inhibitor (MAO-I), is also a good scavenger of LP carbonyls because of the presence of its hydrazine functional group. Phenelzine inhibited oxidative damage and mitigated mitochondrial dysfunction in isolated rat brain mitochondria when subjected to exogenous acrolein or 4-HNE. Further, rats subjected to CCI and given a single dose of 10 mg/kg phenelzine subcutaneously 15 min post-TBI, protected cortical tissue from injury at 2 weeks [168]. Additionally, repeated doses of phenelzine (an initial dose of 10 mg/kg subcutaneous 15 min post-TBI followed by a repeated dose of 5 mg/kg subcutaneous every 12 h up to 60 h post-TBI) protected cortical tissue loss and attenuated mitochondrial impairment in CCI rats [169].

β-Phenylethylidenehydrazine (PEH): PEH is an active metabolite of phenelzine (β-phenylethylhydrazine). Both phenelzine and PEH possess a hydrazine functional group, and therefore, react with LP carbonyls and other LP aldehydes to form hydrazones. Because the compounds have different impacts on MAO inhibition, use of PEH may avoid the drug interactions seen with the use of phenelzine and certain sympathomimetics (tyramine) [170].

### 3.7. Nuclear Factor Erythroid 2-Related Factor 2 (Nrf2)-Antioxidant Response Element (ARE) Pathway

Kelch-like ECH-associated protein 1 (Keap1)-Nrf2-ARE signaling regulates endogenous antioxidant defense system and thus, plays an important role in protecting cells from intrinsic and extrinsic oxidants and electrophiles. Nrf2 heterodimerizes with other transcription factors and binds to ARE leading to expression of ARE-regulated genes that enhance cell survival. Kelch ECH associating protein 1 (Keap1) is a cytosolic repressor of Nrf2, which enhances its proteasomal degradation by binding with it [171]. Following TBI, Nrf2-knockout mice exhibited exacerbated brain injury with increased expression of inflammatory cytokines tumor necrotic factor-α (TNF-α), and interleukins (IL-1β and IL-6), and decreased activity of antioxidant enzymes NADPH: quinone oxidoreductase-1 (NQO-1) and glutathione S-transferase alpha-1 (GST-alpha1) [172].

Nrf2 activators: Sulforaphane (SFN), an Nrf2 activator, resulted in upregulation of the antioxidant enzymes heme oxygenase 1 (HO-1) and NQO-1 and lead to significant reduction in neurological dysfunction, injury volume, and neuronal death in rodent CCI models [173]. Tetra-butylhydroquinone (tBHQ), another Nrf2 activator, reduced nuclear factor kappa B (NF-κB) activation and inflammatory cytokines (TNF-α, IL-1β, IL-6) and attenuated cortical injury and brain edema in closed head-injured mice [174]. In another closed-head mouse model, tBHQ activated Nrf2 and attenuated NADPH oxidase (NOX2) in order to reduce cerebral edema and neurologic deficits [175]. Carnosic acid (CA) activates Nrf2 and in turn upregulates cytoprotective (ARE) genes and inhibits pro-inflammatory genes (through suppression of NF-κB). Early (15 min) and delayed (8 h) administration of CA after CCI in mice, resulted in restored mitochondrial respiration, and reduced neuronal cytoskeletal breakdown [167,176]. In mice subjected to repetitive mild TBI, CA administration improved cognitive and motor functions [177].

Melatonin and N-acetylserotonin (NAS): Melatonin (N-acetyl 5-methoxytryptamine) is a neurohormone with multiple physiological functions and has demonstrated to have anti-inflammatory, antioxidant, and antiapoptotic properties [178,179,180]. NAS, a precursor of melatonin, is a melatonin receptor 1C (MT3) agonist that is also shown to exhibit neuroprotection against TBI in preclinical studies. It exhibits antioxidant properties by directly scavenging oxidants and indirectly acting through antioxidant enzymes [181]. Melatonin might act through Nrf2-ARE signaling, as melatonin treatment in rodents upregulated antioxidant enzymes (HO-1 and NQO-1) downstream to Nrf2, while knockout of Nrf2 partially reversed its neuroprotective effects [182]. Moreover, a double-blinded randomized placebo-control clinical trial is investigating the sublingual melatonin in the treatment of post-concussion syndrome following mild pediatric TBI [183].

N-acetylcysteine (NAC) and N-acetylcysteine amide (NACA): Administration of both NAC and its more BBB-permeable form, NACA, reduced cortical damage in rodents after experimental TBI [184]. NACA treatment, following TBI in rats, activated the Nrf2-ARE pathway, attenuated oxidative stress, and inhibited neuronal degeneration [185]. A systematic review including twenty animal studies and three human trials concluded that although there is sufficient preclinical evidence for neuroprotective effects of NAC/NACA after TBI, well-designed clinical studies are lacking [186].

### 3.8. Endothelial Targeted Antioxidant Enzyme Therapy

Following TBI, the damaged endothelium is a key site for oxidative stress, and the damaged endothelial cells upregulate the expression of cell adhesion molecules, such as Intercellular Adhesion Molecule 1 (ICAM-1). A novel approach is the use of targeted endothelial nanomedicine/antibodies. For example, anti-ICAM-1/CAT is an anti-ICAM-1 antibody conjugated to the antioxidant enzyme CAT. In an experimental model of TBI, anti-ICAM-1/CAT treatment reduced oxidative stress at the BBB and attenuated neuropathological outcomes [187].

## 4. Inflammation

Post-traumatic cerebral inflammation starts within minutes of injury and is characterized by upregulation and secretion of mediators (such as DAMPs, cytokines, and chemokines), infiltration of neutrophils and other myeloid cells, and subsequent glial activation and leukocyte recruitment (Figure 2) [188]. BBB impairment during the acute post-traumatic period allows for the entry of circulating neutrophils, monocytes, and lymphocytes to the injury site and directly influences neuronal survival and death [188,189,190]. The accumulated peripheral and resident immune cells in the brain parenchyma release inflammatory mediators, including but not limited to DAMPs, cytokine, chemokines, ROS, prostaglandins, and complement factors [20], which further potentiates inflammation in the injured brain by recruiting more immune cells to the injury site [191]. However, over time, subsequent production of anti-inflammatory mediators and endogenous protectants suppress both humoral and cellular immune activation (Figure 2). In addition to the infiltrating peripheral blood cells, the resident microglia are activated. These activated microglia help in clearing cell debris and promote tissue remodeling. However, activated microglia also release various neurotoxic substances, such as ROS, RNS, and excitatory neurotransmitters, such as glutamate, that may exacerbate neuronal death [192]. In addition, proliferation and migration of reactive astrocytes, and the development of a glial scar after brain trauma impair axonal regrowth. Overall, complex astrogliosis and the trafficking of immune cells to the injury site can promote tissue repair and neurogenesis via the release of neurotrophic factors, [29] or can exacerbate tissue damage through increased inflammatory mechanisms as well (Figure 2).

### 4.1. Inflammatory Mediators

Within minutes to hours after the injury, damaged cells release many intracellular components, such as heat shock proteins (HSP 60 and 70), nucleic acids, and high mobility group protein B1 (HMGB1) into circulation and the extracellular space [14,193,194]. These released intracellular components act as damage-associated molecular patterns (DAMPs) and activate pattern recognition receptors (PRR) for downstream cell signaling [194,195,196]. In response, astrocytes, microglia, and neurons at the injury site begin secreting cytokines and chemokines [191,197]. In addition to their contribution in immune processes and roles in homeostasis, cytokines also function as messengers of intracellular communication [198], while the chemotactic cytokines, or chemokines, regulate leukocyte activation and migration [199]. These inflammatory signals activate microglia and astrocytes, recruit peripheral immune cells, and increase migration to the site of injury. Once inside the brain, peripheral immune cells secrete large amounts of inflammatory mediators, which add to further tissue damage and remodeling [200,201,202,203]. Many studies have reported upregulated expression of IL-1β, TNF-α, IL-6, CCL2, CCL3, CXCL1, CXCL2, CXCL8/IL-8, CXCL10, CCR2, CCR5, CXCR4, and CX3CR1 within 6 h of TBI [204,205,206]. Similar to animal models of TBI, the levels of many cytokines and chemokines peak at 4 h post-injury in patients [207,208]. Collectively, the previous reports suggest that early upregulation of inflammatory mediators is a robust response to injury that also adds to the subsequent secondary injury and chronic neuropathological processes.

### 4.2. Cellular (Innate and Adaptive) Responses

The inflammatory mediators released post-TBI not only alter the residential CNS cells, but also recruit peripheral cells into the brain. These immune cells polarize toward pro-or anti-inflammatory phenotypes due to surrounding signals from the tissue microenvironment [194,205,209,210]. In cortical impact TBI models, neutrophils are among the first cell types to respond to injury. Upregulated adhesion molecules on vascular endothelium mediate neutrophil entry into the traumatized brain during the early h of the first day post-injury [195,211,212]. Despite the essential role of neutrophil recruitment during peripheral infections and damage, they release huge amounts of ROS and RNS in traumatized brain causing oxidative cellular damage. The presence of neutrophils in the injured brain becomes greatly reduced by 3–5 days post-TBI, and mononuclear leukocytes begin to predominate [195,211,213]. These infiltrated cells are mostly CD45^hi^CCR2^+^Ly6C^+^ monocytes, with a small number of dendritic cells (DCs), T lymphocytes, and natural killer (NK) cells. DCs and T cells infiltrate the brain in lower numbers in a similar fashion as monocytes, and perform specific functions depending on the subpopulations of cells present. DCs are categorized into two- T-cell stimulating conventional dendritic cells (cDCs), and interferon-α secreting plasmacytoid (pDCs) [213,214,215]. Further, T cells are categorized into four sub-types- T helper (T_H_), memory T, cytotoxic T (T_C_), and NK cells, each serve distinct functions. Besides infiltrating peripheral cells, residential microglia and astrocytes simultaneously get activated during early immune response. Activated microglia, along with infiltrating macrophages, phagocytose cellular debris, secrete inflammatory mediators, and add to the local inflammation [211,216,217,218].

Meningeal lymphatic vessels are specialized to facilitate drainage of immune cells and macromolecules into the deep cervical lymph nodes and act as sites for immune surveillance of the CNS [219,220]. Notably, following TBI, activated macrophages may drain into cervical lymph nodes and mediate long-term adaptive response through MHC Class II-dependent antigen presentation [213,221,222]. In agreement, T-lymphocytes get activated and recruited within deep cervical lymph nodes by antigen-presenting cells rather than at the site of CNS injury [223]. Interestingly, HLA-DR, an MHC Class II antigen expressed on macrophages, initiated adaptive immune responses by binding myelin basic protein (MBP) [224,225]. Thus, myelin-loaded macrophages may initiate white matter injury (WMI) post-TBI in a similar way to the autoimmune-mediated demyelination in multiple sclerosis [226]. The fact that pharmacological inhibition of MHC Class II reduced neurodegeneration after TBI [227], therefore, activated macrophages perhaps be a connected link between TBI and chronic adaptive immune responses.

T-lymphocytes do not routinely cross the BBB [228,229] but are functionally diverse subsets that mediate the specific adaptive responses against a presented antigen. In particular, T_H_ cells stimulate antibody production and release cytokines that potentiate activation of macrophages and T_C_ cells. Thus, infiltrating macrophages and T_H_ cells cause neurodegeneration as evident in traumatized brain tissue from animals and TBI patients [227,230,231]. Naïve T_H_ cells differentiate into three T_H_ subtypes, such as T_H_1, T_H_2, and T_H_17 on the basis of clues obtained from secreted cytokines by activated macrophages [209,232,233,234].

Further, T_H_17 cells promoted microglial polarization after experimental autoimmune encephalomyelitis (EAE), while, in vitro, myelin-specific T-lymphocytes induced a pro-inflammatory phenotype in microglia via IL-17 [235,236]. Furthermore, inhibition of T_H_17 influx into the brain protected WM and prevented chronic neurological deficits post-neonatal hypoxia-ischemic injury [237]. In agreement, myelin-reactive T_H_17 cells were found to induce demyelination and to compromise remyelination in animal models of WMI and in multiple sclerosis patients [238,239,240,241]. Moreover, curcumin, an anti-inflammatory compound in the curry spice turmeric, improved TBI outcomes [242] and attenuated activation of T_H_17 in ovalbumin-sensitized mice and in acute graft versus host disease [243,244]. Curcumin further mitigated RORγT-mediated T_H_17 differentiation, decreased MBP-reactive T cells, and attenuated IL-17 secretion by activated T_H_17 cells in EAE [245]. Therefore, therapeutic strategies aiming at T_H_17 production/activity and/or MHC-II inhibition may provide potential possibilities to improve chronic functional outcomes following TBI.

By 10–14 days post-injury, most of the circulating immune cells are largely absent from the injury site. However, F4/80^+^ macrophages and glial fibrillary acidic protein (GFAP)+ astrocytes have been detected at distant sites far from the primary damage. In addition, injured thalamic neurons and WMI have been seen many months after the initial impact and thus indicate an effect of chronic diffuse injury [217,246,247]. More detailed literature on the neuroimmunology of TBI and various components of the post-TBI immune response can be obtained from the recent reviews by McKee and Lukens and Jassam et al. [248,249].

Contrary to previous dogma, cerebral inflammation is now considered to have both injurious and beneficial roles in TBI resolution. The traumatized brain can benefit from inflammation if regulated; otherwise, excessive and chronic inflammatory cascades can take over and contribute to numerous neuropathologies [250]. Therefore, many therapies targeting either specific immune cells or inflammation are gaining interests scientifically and clinically.

### 4.3. Therapies Targeting Inflammation in TBI

Glucocorticoids: Glucocorticoids have broad anti-inflammatory actions. A randomized placebo-controlled trial (Corticosteroid Randomisation After Significant Head injury, CRASH) investigated the effects of an IV corticosteroid (methylprednisolone) infusion within 8 h of TBI in adult patients with a GCS score of 14 or less. They reported higher mortality rates at 2 and 24 weeks in the glucocorticoid-treated patients [251,252].

TNF-α inhibitors: In neuroinflammation, TNF-α induces microglial and astrocytic activation, and influences BBB permeability, glutamatergic transmission, and synaptic plasticity [253]. Treatment with intraperitoneal etanercept, a TNF blocker (repeated every 12 h up to 3 days starting immediately following injury) lowered neuronal and glial apoptosis, attenuated microglial and astrocytic activation, reduced the cerebral damage, and improved cognition and motor ability [254]. Additionally, systemic entanercept adminstration was able to penetrate the contused brain tissue reducing the brain contents of TNF-α, and also stimulated newly formed neuronogenesis [255,256]. Clinically, perispinal administration of etanercept (PSE) has shown early promising results in several studies investigating its effects in neurological recovery and chronic pain post-brain injury [257,258,259,260]. An observational study investigating the role of PSE in chronic management of TBI and stroke patients reported significant improvements of chronic neurological dysfunctions. This beneficial effect was observed irrespective of the duration of ailment with improvements noted in patients treated more than a decade after stroke and TBI [261]. The wide therapeutic window of etanercept makes it a valuable therapeutic tool in the management of patients after TBI. 3,6′-dithiothalidomide, a TNF-α synthesis inhibitor, has also shown neuroprotective effects in experimental mild TBI (mTBI) studies when given up to 12 h after injury [262,263]. However, mice lacking TNF-α showed increased post-traumatic mortality without altering the sequele of TBI pathophysiology, and number of infiltrating cells, suggesting a protective role after TBI [264] and could be modulated wisely to extract better outcomes.

IL-1 inhibitors: IL-1 receptor antagonist (IL-1ra) (Anakinra, recombinant human IL-1ra, rhIL-1ra): Both IL-1α and IL-1β bind to IL-1 receptor type 1 (IL-1r1) and initiate signaling. IL-1ra is an endogenous antagonist of the IL-1r1 and blocks receptor activation by IL-1 [265]. IL-1ra overexpressing transgenic mice showed improved neurological functions with a delayed secretion of pro-inflammatory cytokines in a closed-head injury (CHI) model [266]. A review examined previous experimental studies using anakinra in TBI, elucidated that anakinra has a narrow early therapeutic window with less neuroprotective effects when given at 2 h compared to 5 min or 15 min post-injury [267]. In a randomized controlled phase II trial, 100 mg anakinra was administered subcutaneously once daily for 5 days to severe TBI patients and was observed to be safe, penetrated into plasma and brain extracellular fluid, and modified the neuroinflammatory response. However, only twenty patients were recruited, and so the therapeutic effect of anakinra could not be concluded in the study. Furthermore, considering the evidence from experimental studies regarding the narrow acute therapeutic window for administration of anakinra, it would have been advisable to administer the medication at an earlier time point which could be a limitation of the study due to ethical committee requirements [268].

IL-1β neutralizing antibody (anti-IL-1β antibody, IgG2a/k): Intraventricular infusion of anti-IL-1β antibody (starting 5 min post-injury up to 14 days) reduced cortical microglial activation, minimized neutrophil and T cell cortical infiltration, diminished lesion volume, and improved cognitive function [269]. Further, intraperitoneal anti-IL-1β antibody at 30 min and 7 days post-CCI brain injury in mice reduced the ipsilateral hemispheric edema [270]. It has been reported that TBI leads to activation of NOX2 and subsequent NOD-like receptor family pyrin domain-containing 3 (NLRP3) inflammasome. The activation of NLRP3 inflammasome may lead to recruitment of IL-1 and caspase-1 following TBI [271,272]. Clinically, expressions of IL-18, IL-1β, caspase-1, and apoptosis-associated speck-like protein containing a caspase recruitment domain (ASC) were found to be consistent with poor outcomes after TBI [273], and targeting inflammasome through inhibiting ASC could show a promising result in curbing inflammation in mice [274].

IL-1α inhibitors: IL-1α is another important early mediator of inflammation following acute TBI and its inhibition may be neuroprotective [275]. An experimental research study has investigated the selective and combined inhibition of both IL-1 subtypes in TBI. They investigated the effect of inhibiting IL-1α (IL-1α-deficient mice), IL-1β (IL-1β-deficient mice), and IL-1r1 [IL-1r1-deficient mice and anakinra (IL-1ra)] in cortical FPI. IL-1r1 blockade caused a greater reduction in diffuse cytokine expression compared to individual ablation of IL-1α or IL-1β. Both genetic (IL-1r1-deficient mice) and pharmacological (anakinra) blockade of IL-1r1 protected mice from cognitive dysfunction, which was not seen with selective ablation of both IL-1 subtypes [276]. Therefore, broad targeting of IL-1r1 may be a more effective neuroprotective approach.

IL-6 inhibitors: In experimental mild TBI models, IL-6 and keratinocyte-derived chemokine (KC) were elevated 90 min post-injury, and IL-6 levels correlated with injury levels. This makes serum IL-6 a possible biomarker of TBI severity [277]. In a severe injury, serum IL-6 levels were used as a valid prognostic marker of ICP elevation only in TBI patients but could not be used in patients having both polytrauma and TBI [278]. In one study, mice were subjected to mild TBI, treated with rat monoclonal anti-IL-6 antibodies 10 min after injury, and then immediately exposed to hypoxia for 30 min. Anti-IL-6 treatment reduced brain inflammation and neuronal injury. Additionally, anti-IL-6 antibody administration abrogated the motor incoordination prompted by mTBI and hypoxia [279].

Clinically, there are many medications available for IL-6 inhibition, some of them are monoclonal anti-IL-6 antibodies (siltuximab) or anti-IL6-receptor antibodies (tocilizumab, sarilumab). Thus, IL-6 inhibition has a protective effect following TBI and may be the focus of future extensive TBI studies. Given that IL-1 inhibition improved cognitive but not motor functions and that IL-6 inhibition improved motor coordination, collective inhibition of both IL-1 and IL-6 could be a promising therapeutic avenue for further research.

*HMGB1 inhibitors*

TLR4 inhibitor: HMGB1 is a DAMP and binds to PRRs such as TLR2, TLR4, or receptors for advanced glycation end products (RAGE). As neuronal HMGB1 leads TLR4-mediated secretion of IL-6 from activated microglial/macrophages, either pharmacological inhibition of TLR4 by VGX-1027 or genetic mutation limited post-traumatic edema and inflammation in mice post-TBI [14].

Glycyrrhizin (Gly): Gly is a natural triterpenoid, which possesses antiviral and anti-inflammatory activities and suppresses HMGB1 activities via binding with its HMG box [280]. Previously, glycyrrhizic acid (600 mg/kg, i.p.) was reported to reduce ipsilateral brain edema 24 h post-TBI when administered 15 min prior to injury [14]. Similarly, IV administration of Gly 30 min post-injury downregulates HMGB1-(TLR4/RAGE)-NF-κB inflammatory pathway, resulting in attenuation of brain edema and improvement of motor function in TBI rats [281].

HMGB1 A-box: HMGB1 is comprised of an acidic C-terminus and the DNA-binding domains A-box (contains binding sites) and B-box (pro-inflammatory domain). Therefore, recombinant HMGB1 A-box fragment may compete with HMGB1 for binding to corresponding receptor, and thus, may be exert neuroprotection. In a CCI model of TBI in mice, IV administration of HMGB1 A-box daily for 3 days lowered pro-inflammatory cytokine levels, attenuated BBB breakdown, reduced cerebral edema, and enhanced neurobehavioral outcomes [282].

*Other anti-inflammatory interventions*

Natural anti-inflammatory compounds [curcumin and epigallocatechin-3-gallate (EGCG)]: Curcumin, the active compound in the spice turmeric, decreased glial activation, reduced cerebral edema, and improved neurological functions after CCI injury in mice, possibly by suppressing IL-1β, inhibiting NFκB, and downregulating AQP4 [242]. Natural compounds such as curcumin, which have broad anti-inflammatory effects, may prove to be effective treatments for brain injury since they likely target multiple inflammatory pathways to prevent cell death. For example, EGCG, an antioxidant in green tea, reduced cerebral edema and microglial activation following TBI by lowering expression of AQP4 and GFAP. EGCG further decreased oxidative stress by inhibiting NADPH oxidase activation and increasing SOD activity [283].

Cannabinoids: A fast-growing field of therapeutic intervention in different brain disorders is to modulate the endocannabinoid (eCB) system to harness favorable outcomes [205,284]. Cannabis-based research has come a long way from its introduction in 1838 by William O’Shaughnessy [285] for the treatment of migraines [286,287] and neuropathic pain [285,288,289]. The current cannabis preparations available clinically are Cesamet (nabilone), Marinol (dronabinol: Δ9-tetrahydrocannabinol [Δ9-THC]), and Sativex also branded as Nabiximols (Δ9-THC with cannabidiol) [290]. Sativex has been approved in thirty countries for multiple sclerosis-associated spasticity and central neuropathic pain [291], and for opioid-resistant cancer pain as well [292]. The eCB system is an endogenous system that gets activated by natural cannabinoids or cannabinoid-mimicking substances. The eCB system consists of two main cannabinoid receptors (CB1 and CB2), their endogenous ligands 2-arachidonyl glycerol [(2-AG) and N-arachidonoylethanolamine (anandamide)], and ligand-synthesizing and metabolizing enzymes regulating the secreted ligands [293,294]. The eCB system is essential for cellular homeostasis and physiology, and may have an important contribution in repair processes either after injury or during disease [294,295,296,297,298]. However, various inhibitors to AEA-metabolizing enzyme fatty acid amide hydrolase (FAAH) did not advance to phase III clinical trials as neurological therapeutics [299]. Additionally, an IV cannabinoid analog, dexanabinol (HU-211), has failed to show protective effect after head trauma in phase III clinical trials [300], but the scientific quest continues in the hope for other therapeutic preparations for the treatment of stroke and other brain pathologies [301,302,303]. Treatment with exogenous 2-AG attenuated inflammation, cerebrovascular injury, and subsequent neurological deficits after TBI [304]. Recently, we reported that selective activation of CB2R helped to reduce inflammatory macrophages and thus, protected CBF and behavioral function [205]. Synthetic CB2R agonists such as HU-910 and HU-914 showed enhanced recovery in rodents after closed-head injury [304]. Thus, eCB possesses potential targets for the modulation in diverse TBI pathologies. Exogenous compounds, such as the plant-derived phytocannabinoids or synthetic cannabinoids are being highly incorporated in basic research in TBI therapy. However, full characterization of the eCB system in the settings of TBI and other brain injuries is not fully revealed, but efforts are being made to understand its important part in brain homeostasis.

Remote Ischemic Conditioning (RIC): Most studies involving TBI therapies focus on various drugs that can target secondary injury mechanisms; however, a novel treatment known as RIC involves a non-pharmacological approach [305]. This treatment involves applying a blood pressure-type cuff on the arm, which tightens and loosens, subjecting the limb to short cycles of alternate ischemia and subsequent reperfusion to protect distant organs such as the brain, from any injury [306]. Previously, RIC has been stated to improve CBF [307], possibly through the secretion of humoral factors, such as endothelial NOS, NO, and/or nitrites [308], and anti-inflammatory factors that activate protective pathways against ischemia. These mechanisms may also activate mitochondrial ATP-sensitive potassium channels [309] that can restore the mitochondrial membrane potential and can suppress apoptosis following ischemia-reperfusion [310]. Recently, we reported that RIC improved hematoma resolution in a murine model of intracerebral hemorrhage (ICH) via modulation of AMP-activated protein kinase (AMPK) in macrophages [210]. RIC is gaining popularity, and more and more research has started focusing on this method in an effort to enhance endogenous protection [311,312,313,314,315]. A recent clinical trial on RIC following severe TBI found that RIC significantly decreased levels of blood biomarkers after TBI [313]. However, this clinical trial was relatively small and only looked at biomarkers in patient blood at 0, 6, and 24 h after RIC. Further clinical trials on RIC as a treatment for TBI are desired to evaluate its effectiveness, particularly in regard to patient outcomes, cognition, and standards of living. RIC appears to be a promising and non-invasive treatment for numerous conditions and can be easily combined with other treatments. For example, RIC combined with minocycline was proved to be a safe and low-cost intervention in a mouse thromboembolic stroke model [307]. Moreover, exposing rodents to intermittent and sub-acute RIC upregulated endogenous protection mechanisms limiting secondary injury following TBI [311,312]. Non-invasive approaches, such as RIC, will be better tools to provide neuroprotection after TBI but will still require further testing in clinical settings.

## 5. Programmed Cell Death (PCD)

PCD is another major cause of neuronal cell loss that can continue for days following TBI and is associated with poor prognosis [316]. Important PCD processes include cell cycle activation-dependent cell death, cell death mediated by caspases and pro-apoptotic members of Bcl-2 family, PARP/AIF-dependent death, and calpain/cathepsis-dependent death [317,318]. Understanding these many mechanisms of PCD is essential for developing a therapeutic intervention since multiple mechanisms of cell death are often simultaneously and excessively activated in response to injury. Blocking any one individual cell death mechanism may not be beneficial as other mechanisms can still compensate and lead to cell loss.

### 5.1. Cell Cycle Activation-Dependent Neuronal Cell Death

In several neurodegenerative disorders, markers of cell cycle reentry can be detected long before actual neuronal death, suggesting that mature neurons may re-enter the cell cycle and that these cell cycle events (CCE) may be upstream of neuronal cell death pathways [319]. TBI activates neuronal apoptosis, upregulating cell cycle markers (e.g., Cyclin D1, CDK4, E2F5, c-myc, and PCNA), while downregulating various endogenous cell cycle inhibitors [320,321,322]. Pharmacological inhibitors of cyclin-dependent kinases (CDKs), which regulate the cell cycle, have been stated to attenuate neuronal cell death and significantly improve outcomes after TBI in rodent models [321,323].

### 5.2. Caspase-Dependent Cell Death

Morphological cell changes, such as nuclear fragmentation, chromatic condensation, and membrane budding following TBI, occur via caspase-dependent cleavage of specific apoptotic substrates. Caspase-3 activation can follow either the extrinsic pathway, involving TNF and FAS receptors, or the intrinsic pathway, involving mitochondrial outer membrane permeabilization (MOMP). MOMP induces the release of cytochrome c (cyt c), an inner mitochondrial membranous protein, into the cytosol. There, cyt c binds with apoptosis-inducing factor (Apaf-1) to form an ATP-dependent complex, which in turn activates caspase-9 and caspase-3 [324].

Caspase-3 is an important effector caspase, which plays an important role in injury-induced neuronal loss after TBI [324]. Many studies have reported the link between activation of caspases and neuronal apoptosis in both clinical and pre-clinical TBI [325,326,327,328]. Treatment with various caspase inhibitors improves outcomes after experimental TBI, is effective therapeutically, and has a broad safety window [327,329]. Caspase-12 can be activated by endoplasmic reticulum (ER) stress, triggering apoptosis and resulting in neuronal cell death [328,330]. Bcl-2 and Bcl-xL are anti-apoptotic proteins that either inhibit MOMP directly or inhibit pro-apoptotic proteins to regulate MOMP and caspases. Pro-apoptotic proteins, consist of three subtypes; the first subtype consists of BAX and BAK, which can directly permeabilize mitochondrial membrane; the second subtype includes BID and BIM, which activate the first subtype; and the third type includes BAD, PUMA, and NOXA which can inactivate Bcl-2 and Bcl-xL [318]. The balance between the activities of pro- and anti-apoptotic members of Bcl-2 family is a major determinant of apoptosis after TBI. Increased Bcl-2 and Bcl-xL expression leads to survival of cells [331] while upregulation of BAX, BAD, or BIM promotes cell demise in the post-TBI brain [316].

### 5.3. Caspase-Independent Cell Death Pathways

Following MOMP, mitochondrial inner membrane proteins, such as cyt c, Smac/DIABLO, AIF, and endonuclease G (endoG), may be released into the cytosol and modulate cell death. Calpain I, a Ca^2+^-dependent cysteine protease, is activated following TBI [332,333,334]. Activated calpain I cleaves the death-promoting Bcl-2 family members BID [335,336] and BAX [337], which then translocate to mitochondrial membranes. This results in the release of truncated apoptosis-inducing factor (tAIF) [335], cyt c [338], and endoG [336] in the case of BID or cyt c in the case of BAX [337,338]. These released proteins cause damage to nucleic acids and potentiate the release of inner membrane proteins [339,340,341]. While endoG cleaves internucleosomal (180 base pair) DNA [342], tAIF causes DNA cleavage on a large scale via interaction with phosphorylated histone H2AX (γH2AX) and cyclophilin A after translocation into nuclei [343,344]. Further, activated calpain I cleaves the Na^+^-Ca^2+^ exchanger, which leads to accumulation of intracellular Ca^2+^ [345]. Cyt c has also been shown to translocate to the nucleus and is linked with cytosolic translocation of acetylated histone H2A in irradiated HeLa cells [346,347,348].

Most studies have confirmed that AIF mediates cell death, independent of caspase, Apaf-1, or cyt c [349,350]. PAR polymerase-1 (PARP-1), Cyclophilin A, and HSP-70 are key regulators of AIF and are responsible for translocation of AIF from mitochondria into the nucleus, which is partly mediated by activation of PARP-1 [351,352]. PARP-1 activation causes depletion of cytosolic NAD+ and subsequent mitochondrial dysfunction, which mediates the release of AIF from mitochondria [353,354]. The end-product of PARP-1 activation, poly (ADP-ribose) (PAR) polymer, also cause direct or calpain-mediated mitochondrial impairment and MOMP [355]. PARP-1 is activated in response to DNA damage and forms PAR polymers to repair DNA nicks. However, when DNA loss is extensive, PAR starts building up in the nucleus, and eventually translocates to mitochondria, and causes release of AIF [356,357,358]. Further, nuclear PAR glycohydrolase (PARG) hydrolyzes excessive PAR into ADP-ribose. These ADP-ribose translocate into the plasma membrane to stimulate melastatin-like transient receptor potential 2 (TRPM-2) channels to cause excessive Ca^2+^ influx into neurons [359]. In the main region of injury following cerebral ischemia, where bioenergetics conditions are compromised, cellular death takes place via AIF- and PARP-1-mediated processes rather than caspase-mediated cell death [349]. In fact, in PARP-1-dependent cell death, mitochondria release AIF and cyt c; however, caspases do not become activated because of depleted ATP [353,355].

### 5.4. Therapies Targeting Cell Death Pathways

HSP70: HSP70 is an interesting molecule that is extremely important in neuronal cell survival [360,361]. This mechanism of neuroprotection includes binding of HSP70 to Apaf-1 and AIF, thereby blocking the creation of apoptosome complexes, and subsequent activation of caspase-3 [362,363] and attenuating nuclear translocation of AIF [361,364,365]. In addition, deletion of HSP70 or HSP110 caused increased cell death with upregulated expression of ROS-induced P53-target genes, such as pig1, pig8, and pig12, while HSP70/110 boosting drug celastrol improved behavioral outcomes and protected brain cells from secondary injury following TBI [366]. It must be noted that stress-induced cellular death likely involves multiple pathways [318,367,368]. A key determinant in cell death dynamics in TBI is likely the cellular bioenergetics of the brain. When bioenergetic processes are preserved, caspase-dependent cell death mechanisms predominate, while under deficient bioenergetic conditions, when caspase is not activated, AIF may facilitate cell death. As AIF and caspases act through parallel pathways in apoptosis, targeting both pathways would have potentially additive therapeutic effects [369].

CDK inhibitors: Selective CDK inhibitors inhibit the cell cycle that leads to glial activation and neuronal apoptosis in TBI [370]. These CDK inhibitors are toxic when given chronically, as is done in cancer treatments; however, short-term treatments, such as could be the case for acute TBI, would pose less of an issue. In addition, roscovitine and CR-8 show strong neuroprotective effects when administered as a single dose at a clinically relevant delayed time point [370]. Another TBI study found that CDK inhibitors decreased neuronal death, lesion volume, astroglial scarring, microglial activation, and improved motor and cognition functions [321].

Minocycline: Minocycline is a second-generation tetracycline and has been reported to inhibit microglial activation and subsequent excitotoxicity in TBI [371,372,373,374]. Minocycline also inhibited caspase-dependent and independent mitochondrial cell death pathways by preventing release of cyt c in a chronic neurodegeneration model [375]. However, one study found only transient neuroprotection and no change in apoptosis [189]. Minocycline is already FDA approved as an antibiotic, has a long half-life, can readily cross the BBB, and is well tolerated in high doses [376]. In addition, clinical trials of minocycline treatment in acute spinal cord injury found improvements in neurological outcomes and no significant adverse effects [377]. Therefore, animal studies and clinical trials warrant further investigation of minocycline as a possible therapeutic intervention in TBI.

Progesterone: Progesterone treatment leads to reduced edema, neuroinflammation, neuronal excitotoxicity, and apoptosis after TBI in both animal studies and initial clinical trials [378]. Progesterone modulated AQP4 expression on astrocytes and decreased cerebral edema in rats after TBI [379]. Progesterone may also reduce LP and oxidative stress by upregulating antioxidant enzymes, such as SOD [380], and ROS scavengers, such as mitochondrial glutathione [381]. Progesterone also attenuated neuronal excitoxicity by inhibiting voltage-gated calcium channels [382]. Studies have also shown that progesterone may inhibit activity of cyt c and caspase-3, and upregulate anti-apoptotic Bcl-2 proteins [382]. Despite the widespread success in experimental TBI [378], stage III clinical trials found no significant improvements in progesterone-treated TBI patients [383]. Similar to progesterone studies, many seemingly promising TBI treatments also failed in later clinical trials [384]. For example, erythropoietin restored mitochondrial function in TBI, and thereby reduced oxidative stress and inflammation [385] but failed to show significant improvements in TBI patients in clinical trials [386]. These failures may be attributed to many different factors including mechanistic and physiological differences in animal systems, heterogeneity of the injuries in patients, and/or issues with dosage and durations of treatment [387]. For example, no drug optimization studies were done prior to the phase III progsterone trials even though pre-clinical trials found many parameters that were critical for treatment effectiveness [384]. Studies have stated that growth hormone replacement therapy, in patients with post-traumatic hypopituitarism, partially reversed cognitive impairment, and improved processing speed and memory after TBI [388,389]. Incidence of endocrine insufficiency/failure after TBI is quite high but its cognitive symptoms are often mistaken for signs of residual injury [390]. These hormone deficiencies may be easily treated with hormone replacement therapy, and clinical symptoms respond well to treatment. Greater awareness of hypopituitarism and adrenal and endocrine failure following TBI is desirable in order to better manage the chronic effects of TBI.

microRNAs (miRNAs): Widespread research on therapies utilizing miRNAs, small non-coding RNA molecules that regulate gene expression, is performed in many different disease research fields [391]. miRNAs have demonstrated great therapeutic potential; however, research on these therapies is still in its infancy and the part of miRNAs in secondary injury in TBI remains largely unexplored [392]. Recently, Sabirzhanov et al. found that upregulation of miR-711 in TBI coincided with downregulation of the pro-survival protein Akt and subsequent activation of apoptotic PUMA and BIM, and cytosolic translocation of cytochrome c and AIF [392]. Inhibitor of miR-711 decreased apoptosis, restored Akt, and attenuated long-term neurological deficits after TBI. Another miRNA, miR-21 repressed apoptosis and supported angiogenesis by increasing Bcl-2 expression, inhibiting BAX and caspase-3, and activating PTEN-Akt signaling [393,394]. Going forward, miRNA therapies may be a promising future direction for the development of novel interventions in TBI by enabling direct targeting and inhibition of cell loss and concurrent targeting of multiple effectors.

## 6. Conclusions

In conclusion, a comprehensive understanding of TBI pathophysiology will allow for the development of effective drugs or drug combinations that target multiple secondary injury mechanisms and can be administered during optimal therapeutic windows. This approach could augment the bench to bedside translation of neuroprotective treatments. A few noteworthy treatment strategies have made it to the clinical trial stage for the treatment of brain injury, including tranexamic acid, CDP-choline, methylphenidate, NA-1, CBD, and non-invasive RIC. However, there is still a lack of effective therapeutics for TBI, with treatment mainly consisting of emergency surgeries, maintaining ICP, and rehabilitation therapies. The heterogeneous nature and complex pathophysiology of TBI necessitates a combination of therapies to ameliorate secondary injury and post-traumatic deficits. The fact that neurons and other supporting cells, such as astrocytes, microglia, oligodendrocytes, and the brain vasculature can all undergo degeneration quickly after trauma in the injured brain, further complicates its management. Astrogliosis and neuroinflammation are key secondary injury events that contribute to neurological deficits and even to chronic neurodegeneration after TBI. Moreover, death of non-neuronal cells can compromise recovery and hinder neurotransmission. Future therapeutic strategies should be focused on secondary injury, aiming to minimize detrimental events such as neuroinflammation and create optimal conditions for regeneration and repair post-injury. Additionally, the classification of patients according to GCS score alone may not be an effective method for patient inclusion in clinical trials. Advanced analysis of brain imaging has emerged as a vital tool for identifying progression of disease and efficacy of treatment clinically. Therefore, utilization of novel imaging methods and biomarkers, in addition to GCS scores, may be more accurate criteria for patient recruitment in clinical trials of neuroprotective medications.
